# Comparison of chronic physical and emotional social defeat stress effects on mesocorticolimbic circuit activation and voluntary consumption of morphine

**DOI:** 10.1038/s41598-017-09106-3

**Published:** 2017-08-16

**Authors:** S. E. Cooper, M. Kechner, D. Caraballo-Pérez, S. Kaska, A. J. Robison, M. S. Mazei-Robison

**Affiliations:** 1 Neuroscience Program, Michigan State University, East Lansing, MI 48824 USA; 2 Dept. of Pharmacology and Toxicology, Michigan State University, East Lansing, MI 48824 USA; 3 Dept. of Physiology, Michigan State University, East Lansing, MI 48824 USA

## Abstract

Chronic social defeat stress (CSDS) is a well-established rodent model of depression that induces persistent social avoidance. CSDS triggers molecular adaptations throughout the mesocorticolimbic reward circuit, including changes in the activity of dopamine neurons in the ventral tegmental area (VTA), that may also influence drug reward. One limitation of traditional, physical CSDS (PS) is that injury complicates the study of opiate drugs like morphine. Thus, we sought to characterize a variation of CSDS, termed emotional CSDS (ES), that eliminates this confound. We assessed the effect of PS and ES on mesocorticolimbic circuit activation, VTA gene expression, and morphine intake. We found that PS and ES similarly induced ΔFosB in the hippocampus, but only PS significantly increased ΔFosB expression in the prefrontal cortex and striatum. In contrast, cFos expression was similarly reduced by both PS and ES. Interestingly, we found that PS and ES similarly increased voluntary morphine consumption immediately following stress, despite differences in the magnitude of the depressive phenotype and striatal ΔFosB expression at this time point. Combined, these data suggest that both stress paradigms may be useful for investigation of stress-induced changes in drug behavior.

## Introduction

Chronic social defeat stress (CSDS) is a preclinical rodent model used to study stress-induced mood disorders, particularly depression. It has risen in popularity over the last decade due to its ability to induce anhedonia and social avoidance in mice, and the subsequent sensitivity of these behavioral phenotypes to chronic treatment with typical antidepressant drugs^1–4^. CSDS also induces sensitization to a variety of drugs of abuse, with mice showing alterations in cocaine- and alcohol-associated behaviors^4–7^. While this interaction of stress, mood disorders, and drug sensitization has been well established, the molecular and circuit-level mechanisms contributing to this cross-sensitization are not well understood.

The neurocircuitry driving stress-induced behavioral changes has been largely studied in physical CSDS, and copious evidence supports a pivotal role for reward circuitry in the susceptibility and resilience to both CSDS and sensitized drug responses^5, 6, 8–10^. Indeed, altered activity of dopamine (DA) neurons in the ventral tegmental area (VTA) that project to nucleus accumbens (NAc) is both necessary and sufficient for CSDS-driven social avoidance and reduced sucrose preference^8, 11, 12^. While physical CSDS is well suited for understanding the molecular mediators of stress-induced susceptibility to stimulant drugs such as cocaine, it introduces a number of complications in opiate cross-sensitization studies. For example, physical injury during CSDS may potentiate opiate intake due to the analgesic properties of opiates, which may occlude or mask altered processing of reward. Therefore, we have adopted the emotional CSDS (hereafter, ES) model, in which a mouse observes the physical subordination of another mouse but never physically interacts with the aggressor mouse^13, 14^. This model eliminates the potential caveat of physical wounding, while providing a similar chronic stress timeline. Importantly, ES has been shown to produce similar behavioral changes to physical CSDS (hereafter, PS), such as decreased social interaction and sucrose preference, and also appears to induce similar molecular changes in the VTA, as evidenced by RNA sequence analysis (RNAseq)^13^. Though ES offers many advantages for the study of the interaction between mood-disorders and opiate addiction, complete characterization of the effects of ES on both behavior and reward circuitry signaling is needed to fully interpret such studies.

We performed a side-by-side comparison of PS- and ES-induced effects on immediate-early gene activation, VTA gene expression, and opiate reward and consumption to determine whether ES and PS elicit similar changes. First, we compared ΔFosB and cFos induction following PS and ES. Additionally, we used RT-PCR to validate ES upregulation of VTA genes previously identified with RNAseq^13^. Lastly, we determined the presence of an immediate increase in opiate reward and consumption following both ES and PS, despite differences in social avoidance phenotypes at this time point. These data suggest that the molecular changes occurring immediately after either stressor directly affect opiate sensitization and that mechanisms responsible for long-term neuroadaptations that maintain depressive-like behavior may play less of a role, as morphine preference was normalized two weeks following CSDS. Together, this study demonstrates the similar molecular and behavioral responses to both CSDS paradigms and highlights ES as a useful model in the study of stress-induced changes in opiate reward and intake.

## Results

### Validation of Physical and Emotional CSDS paradigm

**Figure 1:**
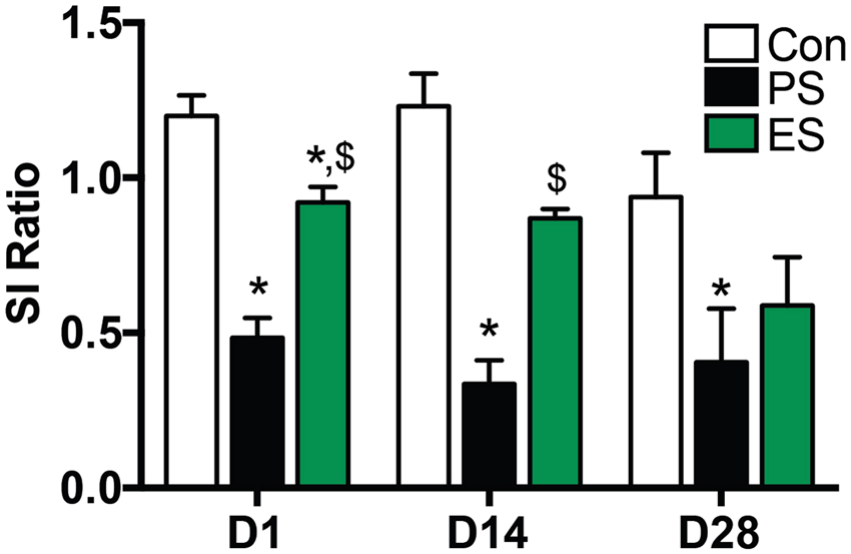
Establishment of chronic social defeat stress (CSDS). Social interaction (SI) was assessed at three time points (Day (D) 1, D14, D28) in separate cohorts following physical (PS) and emotional (ES) stress. D1: control n = 20, PS n = 27, ES n = 24; D14 and 28: control, PS, and ES n = 5 mice/group; *p < 0.05 compared to control, ^$^p < 0.05 compared to PS, Tukey’s post-hoc test.

Male mice were subjected to 10-day physical (PS) or emotional (ES) CSDS and social interaction (SI) ratios were assessed 1, 14, or 28 days post-stress (Fig. 1). Consistent with previous results, there were significant main effects of both stress (F_(2,92)_ = 30.82, p < 0.0001) and time (F_(2,92)_ = 3.276, p = 0.0422), while the stress × time interaction was not significant (two-way ANOVA). Specifically, PS mice had significantly reduced SI ratios compared to controls on D1, D14 and D28, and were also significantly different from ES mice on D1 and D14 (Tukey’s test, *p < 0.05). ES mice have reduced SI ratios compared to controls on D1 (Tukey’s test, *p < 0.05), although they are also significantly different from PS on D1 and D14, illustrating a more modest phenotype. This time-course is similar to published results, where PS mice have a maximal reduction in SI ratio on D1 but ES do not display a similarly robust social avoidance phenotype (~0.6 SI ratio) until D28^13^. Due to the increased variability in both stress groups and controls on D28, and to avoid possible confounds of prolonged social isolation, we used D14 as an intermediate time point for long-term effects in behavioral studies.

### PS- and ES-induced Changes in ΔFosB and cFos in Mesocorticolimbic Brain Regions

It has been shown that PS robustly increases FosB and ∆FosB (a splice variant of FosB) expression throughout the mesocorticolimbic circuit 24 hr following social defeat^15–17^. Additionally, ΔFosB protein levels (western blot) in PS mice correlated with extent of social avoidance in susceptible animals in NAc and PFC^15^. Moreover, direct alteration of ΔFosB levels in NAc and PFC via viral-mediated overexpression was sufficient to alter depressive-like behaviors, supporting at link between ∆FosB expression and depressive-like phenotypes^17, 18^.

**Figure 2:**
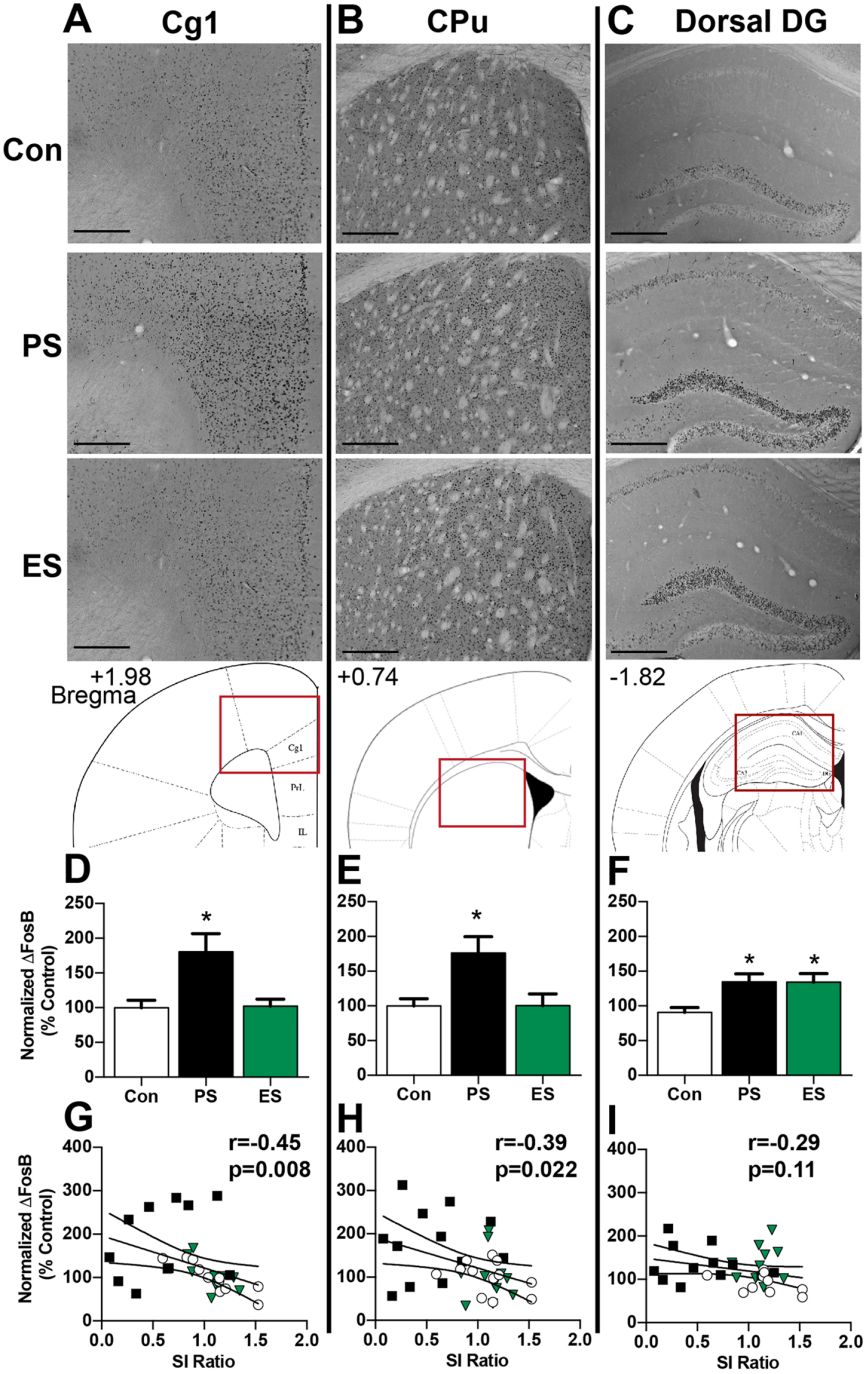
CSDS increases the number ΔFosB-positive cells in select brain regions. Top panel: Representative images of ΔFosB-positive nuclei visualized by DAB-staining in control (Con), physical stress (PS), and emotional stress (ES) mice in (**A**) cingulate cortex (Cg1), (**B**) caudate putamen (CPu), (**C**) Dorsal dentate gyrus (DG) (Scale bar: 200 μm). Schematics of the location of each brain region (red box) within a coronal slice are depicted below (adapted from^36^). (**D**–**F**) Quantification of the number of ΔFosB-positive nuclei, displayed as percent of control. (**G**–**I**) Correlation of ΔFosB counts and SI ratio. Pearson r values and p values are noted on each graph. n = 9–12 mice/group; *p < 0.05 compared to control, Tukey’s post-hoc test.

In order to determine whether PS and ES induce similar changes in brain ΔFosB immunoreactivity, the average number of ΔFosB-positive cells (per mm^2^) was assessed in select brain regions on D1 following standard PS and ES (Table 1). Consistent with previous reports, we found robust increases in ΔFosB expression in the PFC and dorsal and ventral striatum of PS mice, but interestingly, this was not observed in ES mice, where expression was similar to controls (Table 1, Fig. 2A,B). Specifically, there was significantly increased ΔFosB expression in PS mice compared to both controls and ES mice within the Cg1 (Fig. 2A, F_(2,30)_ = 7.039; p = 0.0031, Tukey’s test *p < 0.05) and PrL (control: 100 ± 9.8, PS: 159.9 ± 18.9, ES: 99.2 ± 14.2; F_(2,29)_ = 5.60 p = 0.009, Tukey’s test *p < 0.05) subregions of the PFC, as well as the CPu (Fig. 2B, F_(2,32)_ = 6.252; p = 0.0051, Tukey’s test *p < 0.05), and NAc core (control: 100 ± 4.9, PS: 141.1 ± 14.3, ES: 106.6 ± 10.4; F_(2,30)_ = 4.35 p = 0.022, Tukey’s test *p < 0.05).

**Table 1:** Mean number (+/−SEM) of ΔFosB immunoreactive nuclei per mm^2^ in brain areas of control, physical stress, and emotional stress mice 1 hr after social interaction testing (~24 hours after last day of 10 d of chronic social defeat stress), n = 9–12 mice/group.

	Control	Physical	Emotional	F value
PFC				
Cg1	483 ± 104	731 ± 158*	480 ± 103	F(2,30) = 7.04, p = 0.0031
PrL	409 ± 83	600 ± 134*	435 ± 82	F(2,29) = 5.60, p = 0.0088
IL	390 ± 60	453 ± 85	335 ± 50	F(2,31) = 0.95, p = 0.40
NAc				
Shell	679 ± 223	596 ± 181	613 ± 215	F(2,30) = 0.34, p = 0.72
Core	1278 ± 145	1481 ± 162*	1381 ± 169	F(2,30) = 4.35, p = 0.0219
CPu	257 ± 45	429 ± 73*	309 ± 78	F(2,32) = 6.25, p = 0.0051
VTA	41 ± 9	56 ± 8	43 ± 10	F(2,31) = 1.99, p = 0.15
DR	123 ± 15	181 ± 27	162 ± 26	F(2,30) = 1.43, p = 0.25
Dorsal Hipp				
DG	553 ± 87	724 ± 92*	781 ± 117*	F(2,30) = 4.98, p = 0.0136
CA1	25 ± 6	47 ± 9*	37 ± 9^#^	F(2,31) = 5.38, p = 0.0099
CA3	352 ± 41	412 ± 60	294 ± 39	F(2,32) = 2.01, p = 0.1509

Cg1, cingulate cortex; CA1/3, cornu ammonis 1/3; CPu, caudate putamen; DG, dentate gyrus; DR, dorsal raphe; Hipp, hippocampus; IL, infralimbic cortex; NAc, nucleus accumbens; PFC, prefrontal cortex; VTA, ventral tegmental area.

*p < 0.05, compared to control.

^#^non-significant trend.

Given that we also observe a significant difference in SI ratio between PS and ES mice at this time point, we next completed a correlational analysis to determine the relationship between ΔFosB expression and SI score. We found that ΔFosB counts were significantly negatively correlated with SI ratio in the PFC (Cg1: r = −0.45, p = 0.0084, Fig. 2G; PrL: r = −0.43, p = 0.014, data not shown), such that mice with lower SI ratios (i.e. more “depressed”) had higher ΔFosB expression. This was also the case in the dorsal striatum (CPu: r = −0.39, p = 0.0220, Fig. 2H) and the NAc core displayed a similar trend (r = −0.19, p = 0.28). Together, these data support a link between *FosB* expression in the PFC and striatum and depressive-like behavior, and suggest that the moderate phenotype in ES mice may be related to minimal activation of these brain regions.

While ES did not increase ΔFosB expression in the PFC and striatum, it did increase ΔFosB expression in the hippocampus (Table 1, Fig. 2C). Specifically, we found that there was significantly increased ΔFosB expression in the dorsal hippocampus DG region of both PS and ES mice compared to controls (Fig. 2F: F_(2,30)_ = 4.98 p = 0.0136, Tukey’s test *p < 0.05). In contrast to the PFC and striatum, ΔFosB counts were not significantly correlated to SI ratio in the hippocampus (Fig. 2I). This may suggest that ΔFosB expression in dorsal hippocampus is not linked to depressive-like behavior, but that perhaps other CSDS-related behavioral outputs, such as anxiety-like behaviors, may be driven by hippocampal ΔFosB. We also assessed ΔFosB expression in the VTA and DR as these are major dopaminergic and serotonergic nuclei within the brain and alteration of dopaminergic and serotonergic signaling has been implicated in the etiology of depression. However, we did not observe any statistical differences between groups (Table 1), suggesting that chronic ES and PS do not robustly induce ΔFosB in these brain regions.

We next examined the number of cFos-positive cells in separate tissue sections from the same mice (Table 2). Given the differences in the time-courses of cFos and ΔFosB expression^19^, it is likely that cFos induction is driven by the experience of the social interaction test and not the effect of CSDS *per se*. The pattern of cFos regulation differed from that of ΔFosB in three main ways. Firstly, we observed a general decrease in cFos expression following CSDS, in contrast to ΔFosB expression which was generally increased throughout the mesocorticolimbic circuit. Secondly, we found that exposure to PS and ES always produced similar changes in cFos expression. And thirdly, we did not observe a correlation between SI ratio and cFos expression in any of the regions assessed, suggesting that induction of a depressive-like phenotype was not required for the decreased cFos expression, but that the experience of previous CSDS itself was sufficient.

**Table 2:** Mean number (+/−SEM) of cFos immunoreactive nuclei per mm^2^ in brain areas of control, physical stress, and emotional stress mice 1 hr after social interaction testing (~24 hours after last day of 10d of chronic social defeat stress), n = 9–12 mice/group.

	Control	Physical	Emotional	F value
PFC				
Cg1	92 ± 30	64 ± 16	54 ± 10	F(2,26) = 2.51, p = 0.10
PrL	66 ± 24	66 ± 13	54 ± 11	F(2,26) = 0.23, p = 0.79
IL	36 ± 14	41 ± 12	38 ± 12	F(2,26) = 0.73, p = 0.49
NAc				
Shell	89 ± 24	86 ± 15	70 ± 15	F(2,30) = 0.97, p = 0.39
Core	25 ± 6	17 ± 6	15 ± 4	F(2,29) = 1.03, p = 0.37
CPu	27 ± 9	11 ± 3*	12 ± 5*	F(2,30) = 7.87, p = 0.0018
VTA	41 ± 11	33 ± 10	31 ± 5	F(2,27) = 1.03, p = 0.37
Dorsal Hipp				
DG	71 ± 6	60 ± 5^#^	55 ± 5^#^	F(2,30) = 3.22, p = 0.054
CA1	7 ± 3	2 ± 1	5 ± 1	F(2,30) = 1.48, p = 0.244
CA3	56 ± 13	45 ± 9^#^	38 ± 8*	F(2,30) = 3.76, p = 0.0349

Cg1, cingulate cortex; CA1/3, cornu ammonis 1/3; CPu, caudate putamen; DG, dentate gyrus; Hipp, hippocampus; IL, infralimbic cortex; NAc, nucleus accumbens; PFC, prefrontal cortex; VTA, ventral tegmental area.

*p < 0.05, compared to control.

^#^Non-significant trend.

**Figure 3:**
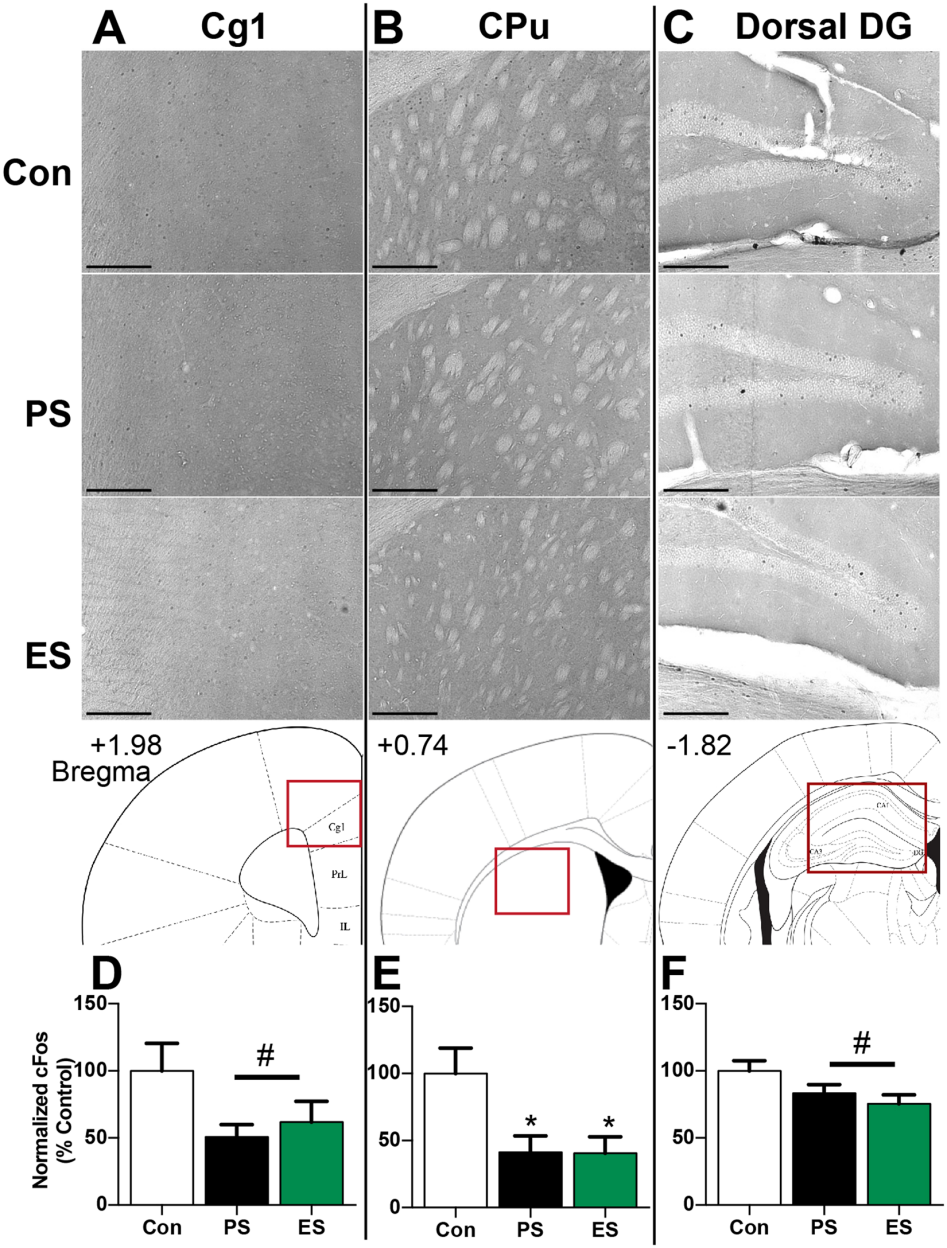
CSDS decreases the number cFos-positive cells in select brain regions. Top panel: Representative images of cFos-positive nuclei visualized by DAB-staining in control (Con), physical stress (PS), and emotional stress (ES) mice in (**A**) cingulate cortex (Cg1), (**B**) caudate putamen (CPu), (**C**) Dorsal dentate gyrus (DG) (Scale bar: 200 μm). Schematics of the location of each brain region (red box) within a coronal slice are depicted below (adapted from ref. 36). (**D**–**F**) Quantification of the number of cFos-positive nuclei, displayed as percent of control. n = 9–12 mice/group, *p < 0.05 compared to control, Tukey’s post-hoc test, ^#^non-significant trend.

However, similar to ΔFosB, we found that the most robust cFos changes were in the PFC, striatum, and hippocampus (Table 2, Fig. 3). While the decreased cFos levels in CSDS mice (compared to controls) in the Cg1 (Fig. 3A,D: F_(2,26)_ = 2.51, p = 0.10) was a nonsignificant trend, the number of cFos-positive cells was significantly decreased in the CPu (Fig. 3B,E: F_(2,30)_ = 7.871, p = 0.0018) and the dorsal hippocampus (CA3) (control: 100 ± 10.3, PS: 80.7 ± 11.1, ES: 59.5 ± 9.7; F_(2,30)_ = 3.761, p = 0.0349) of ES mice compared to controls, with a similar non-significant trend observed in the dorsal DG (Fig. 3C,F: F_(2,30)_ = 3.217, p = 0.0542). We did not observe any significant group differences in cFos expression in the PrL, IL, VTA, or NAc (Table 3).

**Table 3:** RT-PCR analysis of candidate gene expression in the VTA of control, physical stress, and emotional stress mice 24 hr after social interaction testing (~48 hours after last day 10 d of chronic social defeat stress). Data are expressed as fold change +/− SEM, n = 11–25 mice/group.

	Control	Physical	Emotional	F value (from normalized data)
GABRD	1.00 ± 0.09	1.05 ± 0.08	1.02 ± 0.11	F(2,32) = 0.09, p = 0.9150
Kcnj13	1.00 ± 0.16	0.99 ± 0.10	0.98 ± 0.08	F(2,43) = 0.02, p = 0.9842
Prkcd	1.00 ± 0.17	1.105 ± 0.12	0.99 ± 0.11	F(2,32) = 0.2211, p = 0.8029
RAMP3	1.00 ± 0.11	1.00 ± 0.05	0.96 ± 0.07	F(2,32) = 0.1046, p = 0.9010
SGK1	1.00 ± 0.08	2.40 ± 0.36*	2.47 ± 0.41*	F(2,67) = 5.75, p = 0.0050
SGK1.1	1.00 ± 0.05	0.88 ± 0.06	0.90 ± 0.07	F(2,51) = 1.05, p = 0.3579

Overall, we found significant ΔFosB and cFos expression changes within the mesocorticolimbic circuit of both PS and ES mice. Changes in ΔFosB expression were more robust and complex compared to cFos expression, with brain region-specific differences (PFC and striatum) and similarities (hippocampus) observed between PS and ES mice.

### PS- and ES-induced Changes in Voluntary Morphine Consumption

**Figure 4:**
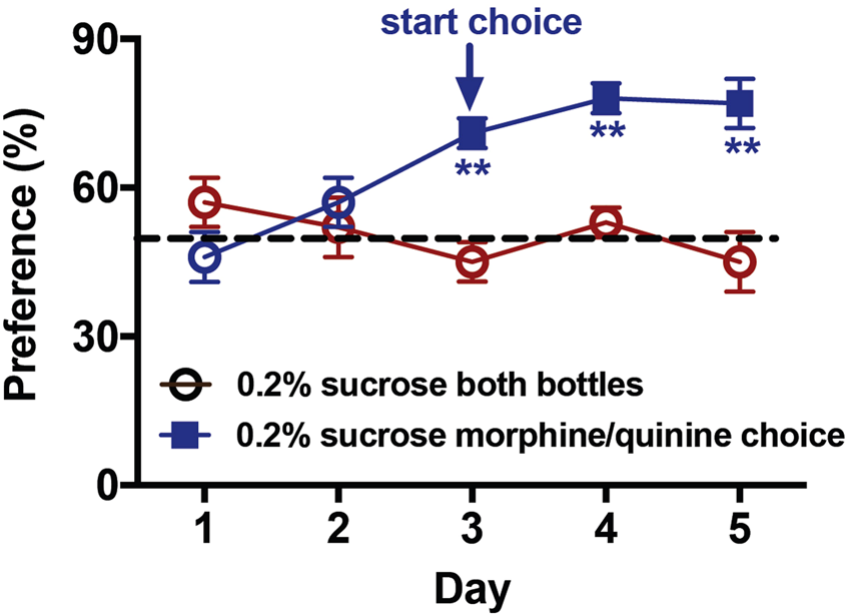
Establishment of voluntary morphine consumption. All mice had access to two bottles containing 0.2% sucrose in their home cage (day 1–2, open circles), and then either continued to have access only to 0.2% sucrose (red open circles) or a choice of 0.3 mg/ml morphine sulfate and 0.06 mg/ml quinine sulfate in 0.2% sucrose (blue squares) on days 3–5. Mice given a choice preferred the morphine solution to the quinine bitter taste control, n = 8 mice/group.

ΔFosB induction in the mesocorticolimbic circuit has been widely studied as a molecular mechanism underlying of aspects of addictive behavior^20^ and given that PS alters drug reward^4–6^, we sought to determine whether ES also affects drug consumption. Given the increase in the prevalence and use of pain-relieving opioid drugs, we chose to assess the voluntary intake of morphine using a two-bottle choice assay. C57Bl6/J mice will voluntarily consume solutions containing morphine^21–23^, and we first established a morphine concentration (0.3 mg/ml morphine) that resulted in a ~75% preference compared to the taste control (0.06 mg/ml quinine sulfate) solution (Fig. 4; Two-way repeated measures ANOVA, Day: F_(4,68)_ = 2.77, p = 0.03, Choice: F_(1,17)_ = 18.35, p = 0.00035, Interaction: F_(4,68)_ = 8.61, p = 0.0001, **p < 0.001, Sidak’s multiple comparison test, choice mice to control mice days 3–5). Importantly, there was no difference in the total intake volume between groups given a choice (morphine/quinine in 0.2% sucrose) and vehicle solution (0.2% sucrose), indicating that the presence of morphine/quinine did not impact overall consumption of fluids (d1-d2 prior to choice: control group = 5.62 ± 0.20 ml, choice group = 5.74 ± 0.23 ml; d3-5 during choice: control group = 5.46 ± 0.14 ml, choice group 5.63 ± 0.22 ml).

**Figure 5:**
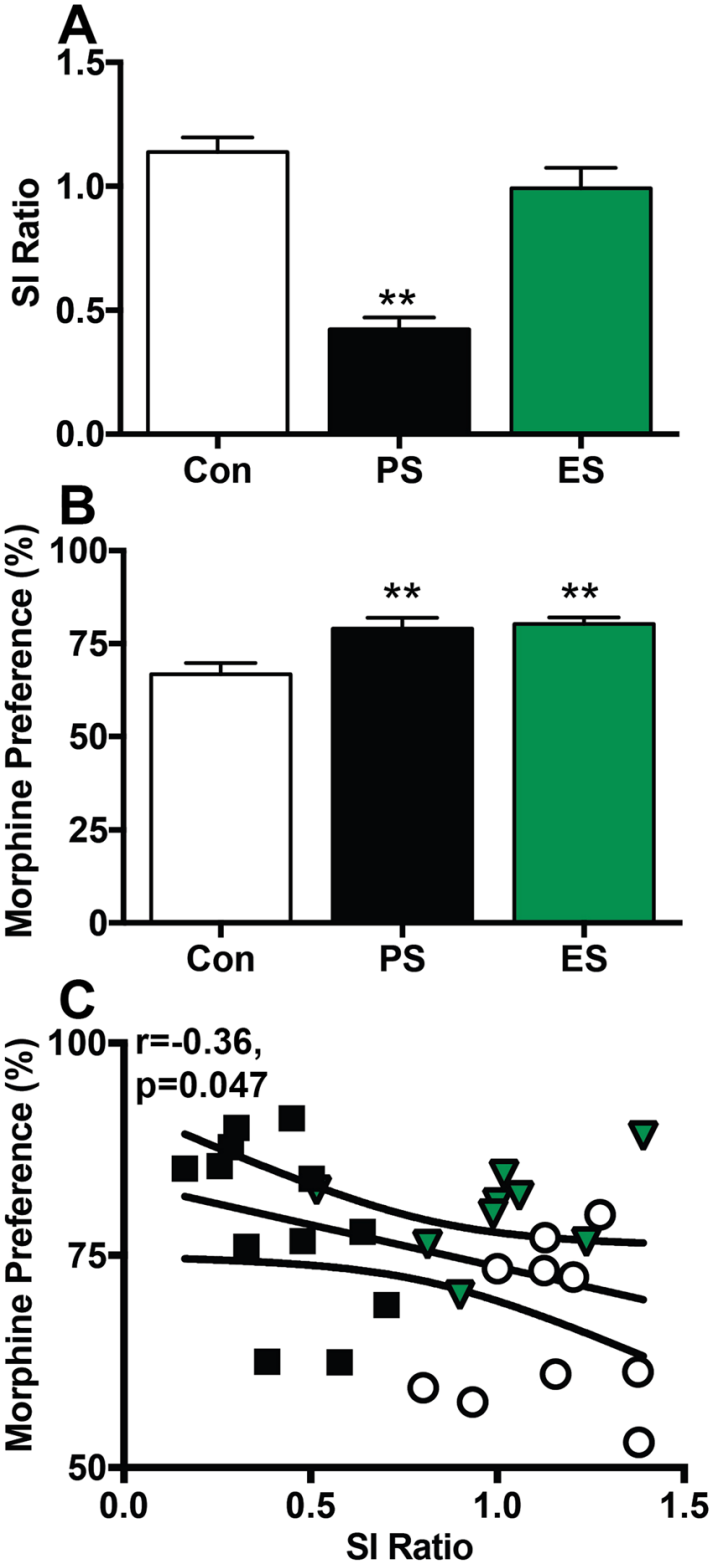
Increased voluntary morphine consumption immediately following CSDS. (**A**) Social interaction (SI) ratio on post-stress day 1 was significantly decreased in physical stress (PS) but not emotional stress (ES) mice. (**B**) Both PS and ES mice displayed significantly increased preference for a morphine solution compared to control mice. (**C**) Morphine preference was significantly negatively correlated with SI ratio. n = 9–12 mice/ group; **p < 0.01 compared to control, Tukey’s post-hoc test.

Next we monitored voluntary morphine consumption following exposure to PS and ES. Within this cohort, at D1, PS mice had significantly reduced SI ratios compared to controls and ES mice (F_(2,28)_ = 40.22, p < 0.0001, Tukey’s test *p < 0.05), while ES mice did not significantly differ from controls (Fig. 5A). While PS and ES groups had significantly different SI ratios, both groups exhibited a similar significant increase in morphine preference compared to controls (Fig. 5B: F_(2,28)_ = 7.265, p = 0.0029, Tukey’s test *p < 0.05). There was also a significant negative correlation between SI ratio and morphine preference (Pearson r = −0.3596, p = 0.047), such that mice with greater social avoidance also displayed higher morphine preference (Fig. 5C). We also examined the total volume of morphine consumed and found that stress increased morphine intake, with ES exhibiting significantly increased intake compared to controls (Control = 3.91 ± 0.32 ml, PS = 4.49 ± 0.22 ml, ES = 4.97 ± 0.19 ml; F_(2,27)_ = 4.1, p = 0.0279, Tukey’s test *p < 0.05). These changes were not dependent on body weight as there were no differences in body weight between groups during morphine choice assays (Control = 22.3 ± 0.5 g, PS = 22.8 ± 0.3 g, ES = 23.0 ± 0.5 g; F_(2,28)_ = 0.76, p = 0.48). These data are consistent with previous work that found increased cocaine CPP in PS-susceptible mice^4^. Moreover, while morphine preference was significantly correlated with SI score, a susceptible phenotype is not necessary to increase morphine reward, as ES mice exhibited increased morphine preference without a significant decrease in SI score. Further, the data suggest that the increase in morphine intake of PS mice is not necessarily dependent on physical trauma or analgesic effects on the resulting pain, as ES, a non-physical stress exposure, was sufficient to increase morphine preference as well.

**Figure 6:**
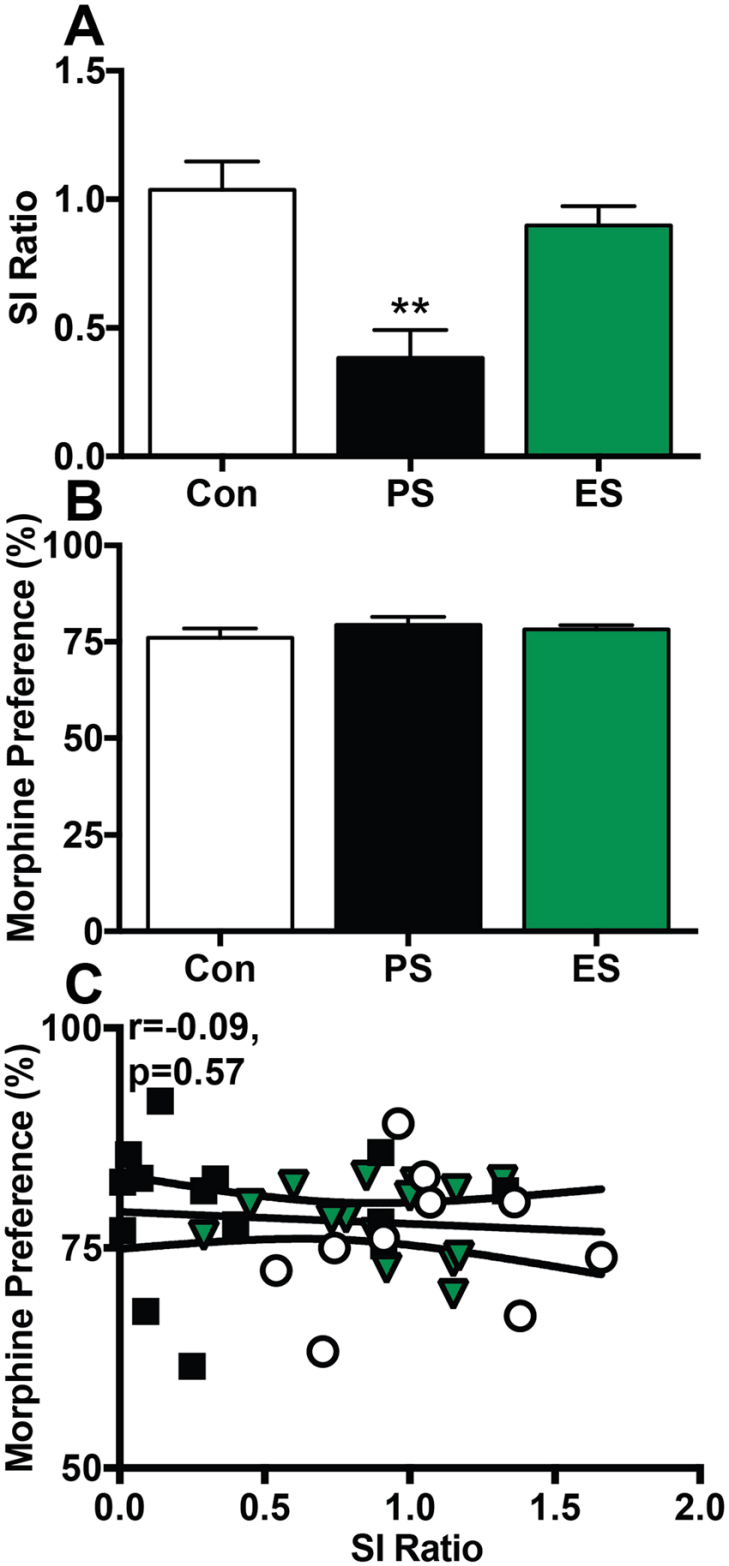
Voluntary morphine consumption is normalized two weeks following CSDS. (**A**) Social interaction (SI) ratio on post-defeat day 14 was significantly decreased in physical stress (PS) but not emotional stress (ES) mice. (**B**) PS and ES mice displayed similar preference for a morphine solution to control mice. (**C**) Morphine preference was not correlated with SI ratio. n = 9–12 mice/ group.

Given that CSDS induces a long-lasting change in SI ratio, we examined whether changes in morphine reward also persist using a second cohort of mice. SI ratio was assessed on D1 and again PS mice had a significant reduction in SI ratio compared to controls, while ES mice did not differ significantly from controls (Control: 1.2 ± 0.05, PS: 0.51 ± 0.13, ES: 1.1 ± 0.05; F_(2,37)_ = 14.23, p < 0.0001, Tukey’s test *p < 0.05). We reassessed SI on D14 before drinking studies began and confirmed similar SI scores to those from D1 (Fig. 6A: F_(2,37)_ = 12.39, p < 0.0001 Tukey’s test **p < 0.01). At this time point, however, the morphine preference did not significantly differ between groups (Fig. 6B: F_(2,36)_ = 0.77, p = 0.47), but we observed a significant increase in morphine intake in PS mice compared to controls (Control = 4.09 ± 0.23 ml, PS = 4.95 ± 0.22 ml, ES = 4.51 ± 0.18 ml; F_(2,36)_ = 3.907, p = 0.0291, Tukey’s test *p < 0.05). The discrepancy between these two measures was likely due to differences in body weight between groups at D14, as PS and ES mice weighed significantly more than control mice (Control = 24.74 ± 0.5 g, PS = 26.6 ± 0.3 g, ES = 26.4 ± 0.3 g; F_(2,36)_ = 6.92, p = 0.0029, Tukey’s post-hoc test p < 0.01), and when intake was normalized to body weight, there was no longer a significant difference between groups (F_(2,36)_ = 2.07, p = 0.14). This difference in body weight was surprising, as previous reports indicate no difference in body weight of PS or ES mice at D28^4, 13^. Together these data suggest that while PS and ES similarly increase morphine reward shortly following defeat, this phenotype is not maintained at later time points, despite the persistence of the depressive-like phenotype.

### PS- and ES-induced Changes in VTA Gene Expression

It has been well established that VTA activity is critical for susceptibility to PS^4, 8, 9^, and changes in VTA gene expression have been implicated as a potential mechanism. Furthermore, we have identified changes in VTA gene expression via RNA-sequencing analysis following chronic morphine exposure, including induction of serum and glucocorticoid-regulated kinase 1 (SGK1), one of the few genes similarly increase by both morphine and cocaine exposure^24^. Therefore, we compared PS- and ES-induced changes in VTA gene expression. Previous RNAseq studies in VTA suggested similar gene expression changes were induced by ES and PS, including induction of SGK1^13^, however changes in the expression levels of specific genes have not been validated. Thus we chose candidate genes (Table 3) from the previously reported dataset.

**Figure 7:**
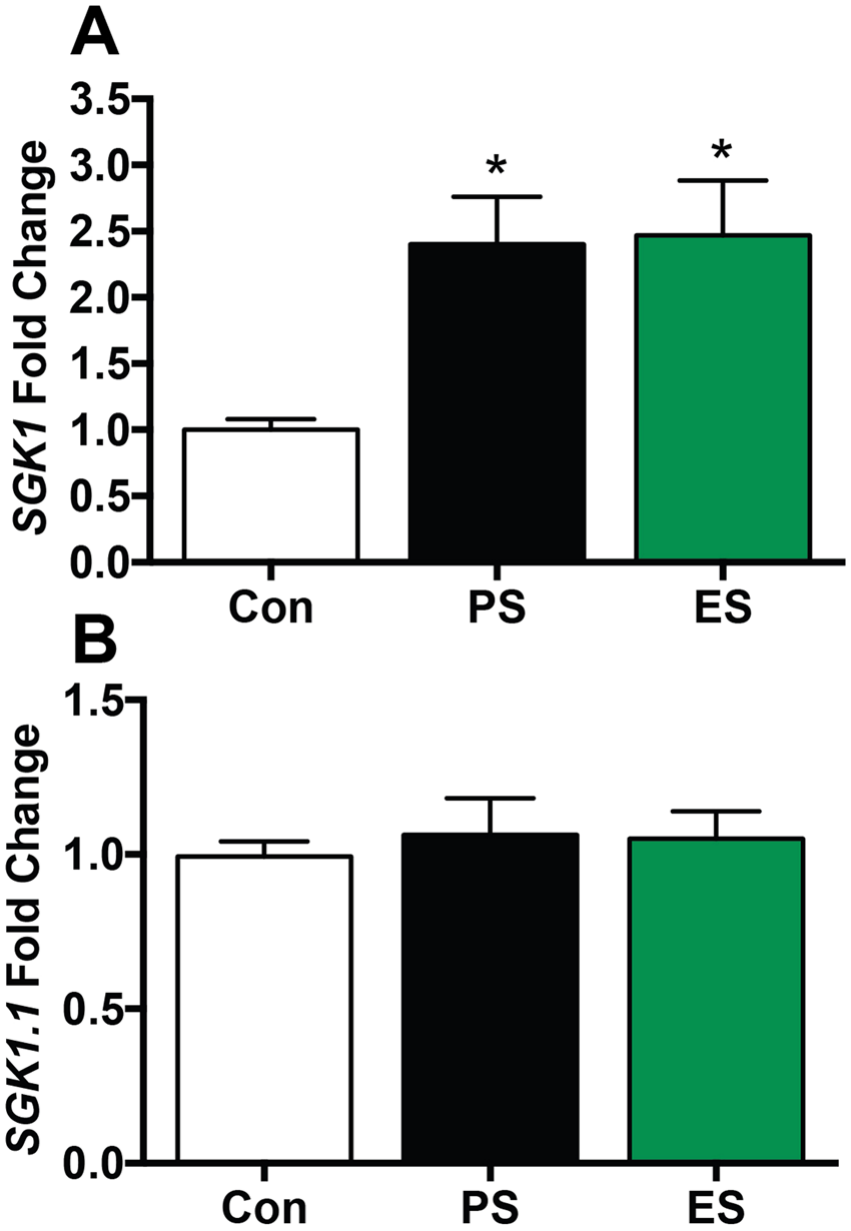
CSDS increased VTA SGK1 gene expression. (**A**) VTA *SGK1* expression was significantly increased in both PS and ES mice compared to non-stressed controls. (**B**) However, expression of the brain-specific SGK1 isoform, *SGK1.1*, did not differ between groups. n = 11–25 mice/ group; *p < 0.05 compared to control, Tukey’s post-hoc test.

Mice underwent PS or ES and VTA tissue was collected for analysis 24 hours following SI testing on D1 as described in previous studies^4, 9, 13^. Using RT-PCR, we confirmed that *SGK1* gene expression was significantly increased in both PS and ES mice (Fig. 7A: F_(2,67) = _5.75, p = 0.0050, Tukey’s test *p < 0.05). However, *SGK1* expression did not correlate with SI ratio (data not shown), suggesting that while SGK1 is induced following chronic stress, it may not drive social avoidance behavior. We also examined SGK1.1, a brain-specific SGK1 isoform known to influence neuronal excitability^25, 26^ and found that *SGK1.1* expression was not significantly altered by stress exposure (Fig. 7B: F_(2,51)_ = 1.048, n.s.) nor did it correlate with SI ratio (data not shown). We also assessed expression of other candidate genes involved in neuronal excitability identified from previous RNA sequencing (*Prkcd*, *Kcnj13*, *Ramp3, Gabrd*), but did not observe any significant differences between groups (Table 3). These data suggest that alterations in VTA gene expression driven by social stress may be subtle, and further work will be required to uncover VTA genes that drive cellular and behavioral responses to stress.

## Discussion

The goal of these studies was to determine whether a variant of CSDS that eliminates physical contact, termed emotional or vicarious CSDS^13, 27^, elicits similar changes in mesocorticolimbic circuitry, reward behavior, and VTA gene expression to those caused by traditional physical CSDS. Similar global changes in VTA gene expression have been previously observed^13^, even at a time point (2 days post-stress) when there are differences in the magnitude of depressive-like phenotypes between PS and ES, suggesting that there may be overlap in the neurobiological underpinnings. Our immunohistochemistry data largely confirm this, as we observed similarly decreased cFos expression in PS and ES mice as well as similar induction of ΔFosB in the hippocampus following both forms of stress. However, we did observe differences in ΔFosB induction between PS and ES in the cortex and striatum, where ΔFosB was significantly increased in PS mice compared to ES mice and correlated with SI score. This suggests that ΔFosB signaling in these regions may be more closely linked with avoidance behavior, with signaling in other areas more indicative of a generalized stress response, and perhaps the anxiety-like phenotype. For example, increasing ΔFosB levels in the PrL region of the PFC is sufficient to increase susceptibility to the social withdrawal phenotype of physical CSDS, without significantly affecting PS-induced anxiety-like behaviors^17^. Moreover, biochemical data show a negative correlation between NAc ΔFosB levels and SI score in susceptible PS mice, where more “depressed” mice have higher NAc ΔFosB protein expression^15^, which is consistent with the increase in NAc ΔFosB-positive cells and correlation with SI score in our current study. However, CSDS-induced changes in NAc ΔFosB expression are complex, as it has also been shown that increasing NAc ΔFosB expression induces resilience to the social withdrawal effects of PS without affecting baseline anxiety-like behaviors, although stress-induced anxiety was not measured^18^. The apparent contradiction between NAc ΔFosB playing a role in both susceptibility and resilience to PS is likely due to induction in separate subsets of medium spiny neurons (MSNs): susceptible mice display increased ΔFosB in D2-type MSNs, while resilient mice show an increase in ΔFosB in D1-type MSNs^16^. Thus, our lack of ΔFosB induction in the PrL and NAc of ES mice is consistent with a modest depressive-like phenotype at this time point, and is also consistent with recent biochemical data that similarly observed no change in NAc ΔFosB protein in ES mice immediately following CSDS^27^. Future studies could potentially explore whether the lack of ΔFosB induction in PFC and striatum of ES mice results from (or perhaps drives) a more subtle depressive phenotype, both by examining a larger cohort of mice with a wider range of SI scores and by virally manipulating ΔFosB expression or DNA binding in ES mice.

In contrast, we found that PS and ES similarly induced ΔFosB in the DG region of the hippocampus. In our immunohistochemistry studies, we did not separately analyze mice susceptible and resilient to PS, as we had a low proportion of resilient mice (2/12 mice) in this cohort. Nevertheless, we observed a significant increase in ΔFosB expression, putting our work slightly at odds with previous data that found ΔFosB expression in the DG was only induced in mice resilient to PS^17^. However, our observation of increased ΔFosB expression in both PS and ES mice is consistent with both models inducing a similar anxiety-like phenotype at this time point^13^. Moreover, the idea of separate mechanisms underlying the depressive- and anxiety-like phenotypes induced by CSDS is supported by work in PS mice, as both susceptible and resilient mice exhibit similar increases in anxiety-like behavior, despite their differences in depressive-like tests (SI and sucrose preference)^4^. Finally, the DG is of particular interest as a candidate for CSDS-induced changes in anxiety behavior given recent data that increasing ΔFosB in mouse DG is sufficient to induce an anxiety-like phenotype^28^. Unfortunately, we were not able to correlate ΔFosB expression with anxiety-like behavior in these studies since we perfused animals shortly after the SI test. However, given the similar magnitude of anxiety-like behavior reported for PS- and ES-mice at this time point, we might predict a positive correlation between these measures and this should be explicitly tested in future studies. Additionally, investigation of whether direct manipulation of ΔFosB expression or activity is sufficient to promote or prevent PS- and ES-induced changes in anxiety-like behavior would be of interest. Overall, our data support the idea that ES induces changes in ΔFosB expression consistent with the PS-induced ΔFosB patterns previously correlated with depressive- and anxiety-like behaviors.

Interestingly, cFos expression was decreased following SI testing in stressed mice. To our knowledge, this was the first study to examine both ΔFosB and cFos changes immediately after SI testing, instead of the typical 24 hour time point following SI testing. Our approach allows a direct assessment of the cFos-derived regional activity caused by social interaction in stressed mice. The cFos data suggest that the pattern of brain activation during SI testing is different between controls and animals that have experienced either PS or ES, but is not dependent on a susceptible, depressive-like phenotype. It would be interesting to investigate whether the decrease in cFos activation in stressed mice also occurred following other behavioral tests, for example assays of anxiety-like behaviors such as elevated plus maze. This might help delineate whether activation changes are specific to exposure to social targets, or are generally induced by exposure to an environment that may induce a physiological stress response. This correlation may also be investigated by measuring stress hormones such as corticosterone, given that mice exposed to CSDS (whether susceptible or resilient) display increased corticosterone and anxiety-like behavior. In contrast to our observation of a general decrease in cFos in the mesocorticolimbic circuit following CSDS in mice, increased cFos has been observed following chronic intermittent stress in rats^29^, an interesting difference given that the models produce other similar neuroadaptations in the mesocorticolimbic circuit^30^. This may be due to procedural differences between the two studies (our study examined cFos 1 day after the last stress episode, immediately following SI testing while the study from Nikulina *et al*. examined cFos 1 week following the last stress and immediately after a saline injection), but further exploration is warranted.

While the role of VTA DA neuronal activity in susceptibility and resilience to CSDS has been well established^11, 12, 31^, we did not observe changes in ΔFosB expression in the VTA of CSDS mice, consistent with previous reports^17^. Although there is evidence that regulation of VTA potassium channels can contribute to the depressive-like phenotype^4, 12, 32^, the molecular mechanisms underlying the increase in VTA DA activity remain largely undefined. One promising candidate for stress-induced adaptations is serum- and glucocorticoid-regulated kinase 1 (SGK1), which is increased in select brain regions following chronic unpredictable mild stress^33^. Indeed, we observe that both PS and ES robustly increase SGK1 gene expression in the VTA, consistent with published RNAseq results^13^. While SGK1 targets in the brain have not been elucidated, SGK1 regulates the expression and activity of a variety of ion channels and transporters in the renal system^34^, suggesting activation of SGK1 in the VTA may play a role in changes in stress-induced changes in cell excitability. Future studies that examine the protein levels of SGK1, as well as direct manipulation of SGK1 expression and activity, are critical to determine what, if any, role VTA SGK1 plays in stress-induced behaviors.

Interestingly, SGK1 gene expression is also robustly increased in the VTA by administration of drugs of abuse, such as cocaine and morphine^24^. CSDS is known to impact drug-related behaviors, with responses generally consistent with increased drug intake, reward, and sensitization. Here, we show that chronic ES also increases drug reward, with ES mice displaying an increase in morphine preference in a two-bottle choice oral morphine assay. Given the established role for NAc ΔFosB in mediating cocaine and morphine CPP^35, 36^, it is interesting that we observe an increase in ES morphine preference without a significant increase in ΔFosB expression. This may suggest that distinct but overlapping mechanisms can drive CSDS-drug cross-sensitization. This is further supported by data that ES and PS mice displayed a similar increase in morphine preference immediately following stress, despite differences in the severity of depressive-like behavior at this time point. However, given previous data the mice resilient to PS do not display changes in cocaine CPP, it suggests that exposure to CSDS alone is not sufficient to alter drug reward, and that induction of at least a modest depressive-phenotype may be necessary. Together, the ability of both ES and PS to increase morphine preference and intake is consistent with the broader literature that exposure to a variety of acute and chronic stressors is sufficient to increase opiate reward, intake and reinstatement in rodents^37–41^, consistent with reports of stress-associated increases in craving in humans^42^. However, the data available on stress-induced changes in opiate reward and self-administration is much more limited than that of cocaine, and some differences have been noted. For example, social defeat stress has been shown to promote escalation, or “binge” self-administration of cocaine, but not heroin, in rats^43^, consistent with data that neurobiological mechanisms underlying opiate and psychostimulant addiction can be distinct^44^, and that findings generated using stimulant models may not translate to opiate drugs.

When we examined morphine intake 14 days following PS and ES, morphine preference of stressed mice no longer differed from controls, suggesting that long-term changes in neural activity and/or gene expression that maintain the depressive-like phenotype are not sufficient for a persistent change in morphine reward in adult mice. While a significant amount of work has focused on the role of stress-induced changes in mesocorticolimbic circuit function in drug responses, much remains unknown about the molecular mechanisms responsible and their persistence, partially due to the variety of stress models and species studied^7^. Thus, identification of candidate mechanisms induced in multiple stress models, such as VTA SGK1, are critical for future studies in order to better characterize the effects of stress on both the depressive-like phenotype and on sensitized drug responses and eventually translate findings to humans.

## Methods

### Animals

Adult (8–9 wk) male C57BL/6J mice (Jackson Laboratory) and CD1 retired breeders (Charles River) were habituated to the animal facility > 1 week prior to experiments. All animals had *ad libitum* access to standard chow and water and were kept on a 12 hr light-dark cycle. All experiments were approved by the Michigan State University Institutional Animal Care and Use Committee (IACUC) and were carried out in accordance with the guidelines set in the Guide for the Care and Use of Laboratory Animals of the National Institutes of Health.

### Physical and Emotional Chronic Social Defeat Stress (CSDS)

Physical and emotional CSDS were performed as reported previously^14, 45^. Briefly, retired male CD1 breeders were initially screened for consistent home-cage intruder aggression before use as aggressors in CSDS. Experimental mice (C57BL/6J) were either placed into the home cage of a CD1 aggressor (physical stress, PS), or placed on the opposite side of the perforated plexiglass divider where they could witness the aggressive encounter (emotional stress, ES) for 8–10 min. PS and ES mice were then separately co-housed with a CD1 aggressor, but kept physically separated from the aggressor via a perforated divider for the remaining 24 hr, allowing for continued sensory stress. PS and ES mice were exposed to a new CD1 aggressor daily, for 10 consecutive days, and all behavioral experiments were carried out between 8:00 and 11:00 AM.

Non-stress C57BL/6 J control mice (Con) were co-housed with each other in standard cages but physically separated by a perforated plexiglass divider. This allows for physical isolation with continuous sensory contact similar to that experienced by PS and ES mice. Control mice were exposed to a novel conspecific daily for the 10-day experiment period.

### Social Interaction (SI) Testing

Social interaction (SI) testing was used to designate mice as susceptible or resilient to CSDS following standard procedures^14, 45^. Following the 10^th^ CSDS exposure, mice were singly housed in standard cages for 24 hr prior to SI testing on post-defeat day 1 (D1). SI was also assessed at later time points (D14 and D28) in some experiments, with mice remaining singly housed for the duration of the experiment prior to SI testing. The social interaction arena (42 cm × 42 cm) contained a cylindrical Plexiglas/wire-mesh enclosure located in the center of the back wall. The interaction zone was defined as an 8 cm region surrounding the cylinder. Locomotor activity and time spent in the interaction zone were quantified by video tracking software (TopScan Suite, CleverSys) for 2.5 min of free exploration (target absent), followed by a 2.5 min session with the CD1 target present in the wire-mesh enclosure. SI ratio was calculated as: (time spent in the interaction zone, target present)/(time spent in the interaction zone, target absent). Scores < 1.0 were defined as “susceptible,” and scores ≥ 1.0 were defined as “resilient”^4^.

### ΔFosB and cFos Immunohistochemistry (IHC)

Physical and emotional CSDS was performed as described above. Mice underwent intracardial perfusion with 4% paraformaldehyde one-hour following SI testing on D1, and brains were post-fixed for 24 hr, cryoprotected in 30% sucrose and stored at 4 °C, per standard procedures^15, 46, 47^. Brains were sectioned (coronal, 35 µm), and selected brain regions were identified according to Paxinos G^48^. Regions examined included: three subregions within the prefrontal cortex (PFC): cingulate cortex (Cg1), prelimbic cortex (PrL), and infralimbic cortex (IL); two subregions within the nucleus accumbens (NAc), shell and core; three subregions within the dorsal and ventral hippocampus (HIPP): dentate gyrus (DG), *cornu ammonis* 1 (CA1) and *cornu ammonis* 3 (CA3); the ventral tegmental area (VTA); the caudate putamen (CPu); and the dorsal raphe (DR).

Immunohistochemistry and quantification of ΔFosB- and cFos-positive nuclei were performed in separate experiments following published protocols^15, 49^. Sections were incubated overnight at room temperature with primary antibody: mouse monoclonal anti-FosB (Abcam, ab11959) or goat polyclonal anti-cFos (Santa Cruz, sc52), both 1:1000, prepared in 3% normal donkey serum and 0.3% Tween-20-PBS. Immunoreactivity was visualized using 3-3′-diaminobenzidane (DAB)-peroxidase staining with biotinylated secondary antibodies (Biotin-Sp-conjugated Donkey-Anti-Mouse or Donkey-Anti-Goat (Jackson) at 1:100) and VECTASTAIN Elite ABC HRP kit (PK6100). ΔFosB- and cFos-positive nuclei were counted by an experimenter blind to experimental conditions on a light microscope (Olympus BX53) using a 40x dry objective. Results are presented as average total cells/mm^2^ per mouse ± SEM (Tables 1 and 2). To allow for a similar scale between brain regions, data in Figs 2 and 3 and in text are presented as percent of control mice.

**Note:** Immunohistochemistry for ΔFosB was performed using an antibody that recognizes full-length FosB, ΔFosB, and Δ2ΔFosB, as well as other Fra antigens^15^. Therefore, we cannot attribute the changes we observe in staining to any specific *FosB* gene product. However, we chose to use the term ΔFosB throughout the text for simplicity, and because ΔFosB was the predominant isoform observed by Western blot in similar experiments performed by our group in the past^15^.

### Real-time polymerase chain reaction (RT-PCR)

PS and ES was performed as described above, and mice were sacrificed 24 hrs following SI testing and VTA tissue was microdissected (14-gauge midline punch) and stored at −80 °C until RNA processing following standard protocols^50^. RNA was extracted using RNeasy micro columns (Qiagen) and reverse-transcribed with a high capacity cDNA reverse transcription kit (Applied Biosystems). Expression changes of candidate genes were assessed via RT-PCR using SYBR green (CFX connect, BioRad). Samples were run in triplicate and normalized to glyceraldehyde-3-phosphate dehydrogenase (GAPDH) using the ∆∆Ct method^3^. Forward and reverse primer pairs are listed in Table 4.

**Table 4:** PCR primers for RT-PCR analysis.

	Forward primer	Reverse primer
GABRD	AACCTGGATGGCTTAATGG	CTCGTTGGTATGGTTGTAGG
GAPDH	AGGTCGGTGTGAACGGATTTG	TGTAGACCATGTAGTTGAGGTCA
KCNJ13	CCACTGGCTTFTCTTTGCAG	GGCAAGTAAGGCGATTGCAC
PRKCD	CAGTATTTCCTGGAGGATGG	TGCACACACGAACAGAAGG
RAMP3	GCTGCTTTGTGGTGAGTGTG	TGATGTTGGTCTCCATCTCGG
SGK1	ATCGTGTTAGCTCCAAAGC	GTCTGTGATCAGGCATAGC
SGK1.1	ATCGTGTTAGCTCCAAAGC	GAAGGCGGATCGGGATACAGATGCA

### Morphine Drinking Studies

Voluntary drinking was assessed by a two-bottle choice test using 50 ml plastic conical tubes fitted with standard sipper tops following established procedures^51^. Briefly, bottle placements (left or right side of standard home-cage) were switched daily to account for individual side-preference. Total fluid consumption was measured daily by changes in total bottle weight and by marking of fluid levels on each bottle. Habituation to the bottles and basal fluid consumption was assessed using tap water 1–2 days prior to morphine choice experiments. Morphine sulfate was kindly provided by the NIDA Drug Supply Program and quinine hemisulfate salt was purchased from Sigma (22640).

To determine a morphine concentration which would elicit ~75% morphine preference in non-stress control C57Bl/6 J mice, we conducted a pilot study using naïve male mice. Based on published results^22, 23^, bottles contained either 0.3 mg/ml morphine or 0.06 mg/ml quinine (as a bitter taste control) in 0.2% sucrose. Fluid consumption is reported as either the mean fluid intake (ml) or morphine preference calculated as (morphine solution consumed/total fluid consumed) × 100. To determine if morphine consumption changed over time, morphine preference and consumption were assessed on D3-6, or D16-19 in separate cohorts. The average intake or morphine preference over 4 consecutive days is reported.

### Statistical Analyses

All data are expressed as mean ± SEM. Statistical significance was determined using two-way analysis of variance (ANOVA) or one-way ANOVA, followed by post-hoc Tukey’s test when appropriate. Correlational analysis used the Pearson’s r test. P values < 0.05 were considered statistically significant.

### Data Availability

All data generated or analyzed during this study are included in this published article.
